# Exergames Encouraging Exploration of Hemineglected Space in Stroke Patients With Visuospatial Neglect: A Feasibility Study

**DOI:** 10.2196/games.7923

**Published:** 2017-08-25

**Authors:** Bernadette C Tobler-Ammann, Elif Surer, Eling D de Bruin, Marco Rabuffetti, N Alberto Borghese, Renato Mainetti, Michele Pirovano, Lia Wittwer, Ruud H Knols

**Affiliations:** ^1^ Physiotherapy and Occupational Therapy Research Center Directorate of Research and Education University Hospital Zurich Zurich Switzerland; ^2^ Care and Public Health Research Institute (CAPHRI] Maastricht University Maastricht Netherlands; ^3^ Graduate School of Informatics Department of Modeling and Simulation Middle East Technical University Ankara Turkey; ^4^ Applied Intelligent Systems Laboratory Department of Computer Science Università degli Studi di Milano Milan Italy; ^5^ Institute of Human Movement Sciences and Sport Department of Health Sciences and Technology Eidgenössische Technische Hochschule Zürich Zurich Switzerland; ^6^ Polo Tecnologico Istituto di Ricovero e Cura a Carattere Scientifico Fondazione Don Carlo Gnocchi Onlus Milan Italy; ^7^ Parkinson Center, Epileptology Neurorehabilitation Clinic Bethesda Tschugg Switzerland

**Keywords:** exergames, eye tracking, virtual reality, visuospatial neglect, feasibility

## Abstract

**Background:**

Use of exergames can complement conventional therapy and increase the amount and intensity of visuospatial neglect (VSN) training. A series of 9 exergames—games based on therapeutic principles—aimed at improving exploration of the neglected space for patients with VSN symptoms poststroke was developed and tested for its feasibility.

**Objectives:**

The goal was to determine the feasibility of the exergames with minimal supervision in terms of (1) implementation of the intervention, including adherence, attrition and safety, and (2) limited efficacy testing, aiming to document possible effects on VSN symptoms in a case series of patients early poststroke.

**Methods:**

A total of 7 patients attended the 3-week exergames training program on a daily basis. Adherence of the patients was documented in a training diary. For attrition, the number of participants lost during the intervention was registered. Any adverse events related to the exergames intervention were noted to document safety. Changes in cognitive and spatial exploration skills were measured with the Zürich Maxi Mental Status Inventory and the Neglect Test. Additionally, we developed an Eye Tracker Neglect Test (ETNT) using an infrared camera to detect and measure neglect symptoms pre- and postintervention.

**Results:**

The median was 14 out of 15 (93%) attended sessions, indicating that the adherence to the exergames training sessions was high. There were no adverse events and no drop-outs during the exergame intervention. The individual cognitive and spatial exploration skills slightly improved postintervention (*P*=.06 to *P*=.98) and continued improving at follow-up (*P*=.04 to *P*=.92) in 5 out of 7 (71%) patients. Calibration of the ETNT was rather error prone. The ETNT showed a trend for a slight median group improvement from 15 to 16 total located targets (+6%).

**Conclusions:**

The high adherence rate and absence of adverse events showed that these exergames were feasible and safe for the participants. The results of the amount of exergames use is promising for future applications and warrants further investigations—for example, in the home setting of patients to augment training frequency and intensity. The preliminary results indicate the potential of these exergames to cause improvements in cognitive and spatial exploration skills over the course of training for stroke patients with VSN symptoms. Thus, these exergames are proposed as a motivating training tool to complement usual care. The ETNT showed to be a promising assessment for quantifying spatial exploration skills. However, further adaptations are needed, especially regarding calibration issues, before its use can be justified in a larger study sample.

## Introduction

Unilateral spatial neglect (USN) is characterized by the inability to detect, respond, or orient toward stimuli presented on the contralateral side of a brain lesion [1]. Being a neurological disorder of attention, USN can affect the auditory, visual, or motor system [2-4]. With 43% in the acute phase and 17% at 3 months poststroke, USN is the most common and persistent problem associated with lesions of the right temporoparietal cortex [5]. Furthermore, USN patients share an unawareness of their deficits to different extents. This anosognosia, combined with an associated reduction in the ability to cope with activities of daily living [6], typically results in longer rehabilitation periods [7-10]. Therefore, USN is a predictor of poor outcome in stroke patients and an added burden for the health care system [7,8,11] requiring efficient treatment modalities [2,12-14].

A variety of accepted and proven traditional methods exist to treat USN [2,15-19], such as pharmacological interventions [20], different physiological sensory stimulations [21-23], and cognitive behavioral training [24]. A combination of multiple approaches to develop a personalized rehabilitation process is recommended [25,26] together with use of a battery of tests to assess USN rather than a single sole assessment [14,27]. However, none of these traditional methods could completely rehabilitate the condition, and rehabilitation methods investigating new approaches are warranted.

Virtual reality (VR), defined as “an advanced form of human-computer interface that allows the user to ‘interact’ with and become ‘immersed’ in a computer-generated environment in a naturalistic fashion” [28] shows some preliminary evidence favoring its use, and further investigations in stroke rehabilitation may complement traditional USN treatment methods [29-32]. VR methods provide a safe copy of the real environment while allowing the creation of customized rehabilitation programs through progressive, repetitive training with immediate feedback [13,19,26,33,34]. Promising VR instruments exist both for the assessment [35-39] and rehabilitation [30-32,40-44] of neglect [26]. The VR assessments were not only able to accurately detect USN patients but also made USN-related symptoms visible that were previously not identified with conventional assessments [26,33,34]. The VR systems tested for rehabilitation, however, were mostly complex to set up or used rather expensive tools (eg, head-mounted displays or cyber gloves), restricting their use to laboratory settings.

The European research consortium Rehabilitative Wayout in Responsive Home Environments (REWIRE) developed a nonimmersive VR system for stroke patients using portable devices with good performance and affordable equipment [45]. By creating such a VR system, REWIRE aimed to facilitate its use for stroke patients discharged from the hospital to allow continuation of the rehabilitation process within their own homes. A variety of home-based VR systems already exist for stroke patients mainly focusing on motor recovery [46-49], but none exist for USN. Therefore, the consortium designed, among others [50], exergames for the treatment of visuospatial neglect (VSN) (VSN being a subtype of USN [4]) [41,51]. Exergame is a portmanteau of the words exercise and game [52], allowing the patients to exercise their skills through gaming. In contrast to games that are designed for diversion for healthy persons, exergames should follow therapeutic principles—for example, the principles of exercise training, such as specificity and progression [53], or adopting the training method of shaping [54], including frequent feedback and the selection of tasks addressing the individual deficits of patients. The REWIRE consortium adopted the principles of exercise training as described by Hoffman [53] to design the neglect exergames. Therefore, the games include the principles of (1) specificity, implying that the required performance of each game corresponds to the goal of the game (to explore the neglected space) and (2) overload and progression, stating that the components being used must be exercised at a level the patient is not normally accustomed to and the patient should progress once accustomed to a level. In order to quantify training progression from simple to complex within each game, the REWIRE consortium used Gentile’s taxonomy of motor skills as a template to develop the exergames (see Borghese et al [55] and Wüest et al [56] for more detail). Due to the nature of neglect and the related unawareness of neurological deficits [4], it is important to test such a novel intervention with the target patient population in a surrounding where close monitoring is possible and feasibility of the approach can be tested. Feasibility may cover aspects such as adherence, safety, and attrition to the novel intervention or whether the intervention and assessments all run smoothly [57].

Mainetti et al [41] and Sedda et al [32] already tested a former version of the REWIRE VSN exergames in a single-case study design involving a neglect patient in the chronic stage. The results were promising in terms of a positive attitude of the patient toward the exergames and in showing a trend for improvement of the VSN-related deficits in daily life. Based on the experience with the exergames of this single user together with feedback on their usability, the exergames were adapted and improved and then tested in this study for the first time in a case series of patients. We aimed to test the exergames in early stroke patients shortly before their discharge to home. This time point was chosen to include as realistic a target population as possible while still guaranteeing safety and supervision of the patients playing the exergames in the supportive environment of the rehabilitation clinic. Specific aims were to determine the feasibility of the exergames with minimal supervision in terms of (1) implementation of the intervention, including adherence, attrition, and safety, and (2) limited efficacy testing, aiming to document possible effects on VSN symptoms in patients early after stroke.

## Methods

### Study Design

We adopted a quasi-experimental pretest-posttest design with a subsequent follow-up to test the feasibility of the exergames in a case series of stroke patients with VSN symptoms. As we aimed to assess implementation of the exergames, thus testing if our intervention can be fully implemented as planned and proposed, an uncontrolled pretest-posttest design is appropriate [57]. A broad variety of definitions exist for the concept of case series in literature [58]. For our study, we used the definition of a case series as being a “report on a series of patients with an outcome of interest” [59]. Recruiting a small convenience sample was ideal for the planned limited efficacy testing, as we aimed to gain intermediate rather than final outcomes in this feasibility project, which allowed us to plan a shorter follow-up period [57].

### Patients

Identification of potential patients for this project was carried out by staff neuropsychologists and occupational therapists in 2 collaborating rehabilitation clinics (Klinik Bethesda Neurorehabilitation, Parkinson-Zentrum, Epileptologie, Tschugg, Bern, and Zürcher RehaZentrum Wald, Faltigberg-Wald, Zurich). They screened all incoming stroke patients with a diagnosis of VSN for eligibility in this study. We aimed for at least 5 participants, as this amount is considered the minimum reasonable number of independent subjects in a group to combine their data [58]. Fewer than 5 patients are usually presented in a descriptive, narrative form of individual case reports. We strived for a maximum of 10 patients as recommended by Abu-Zidan et al [58]. We included patients with a right brain lesion (RBL) due to a first stroke 15 to 180 days after the cerebral event and a diagnosed VSN as measured by the Catherine Bergego Scale (CBS) [60]. Inclusion criteria were being able to sit in a chair or wheelchair with a backrest for 45 minutes, being at least 18 years old, and having a clear view (with or without vision aids) of a computer screen placed at a distance of 60 to 65 centimeters from patient’s face. VSN patients were excluded if their neglect was diagnosed as due to brain injury other than stroke, if severe apraxia was present—measured as less than 5 points on the Apraxia Screen of TULIA (test for upper limb apraxia) (AST) [61]—or if other noncontrolled medical conditions (eg, chronic pain, drug abuse) were present. Patients with a left brain lesion due to a first stroke were excluded because the exergame difficulty levels were designed to progress from the right (easy) to the left (difficult) side of the computer screen. An option to run the games vice-versa (from left to right) was not available.

All patients signed written informed consent before study entry. Ethical approval for the study was received from the local ethics committees (Zurich No. 2014-0543 and Bern No. 389/2014) as well as from the Swiss agency for the authorisation and supervision of therapeutic products (Swissmedic, 2015-MD-0003). The latter approval was required as the software was not yet certified with the European Community marking for medical devices. The study is registered at ClinicalTrials.gov [NCT02353962].

### Setup

**Figure 1:**
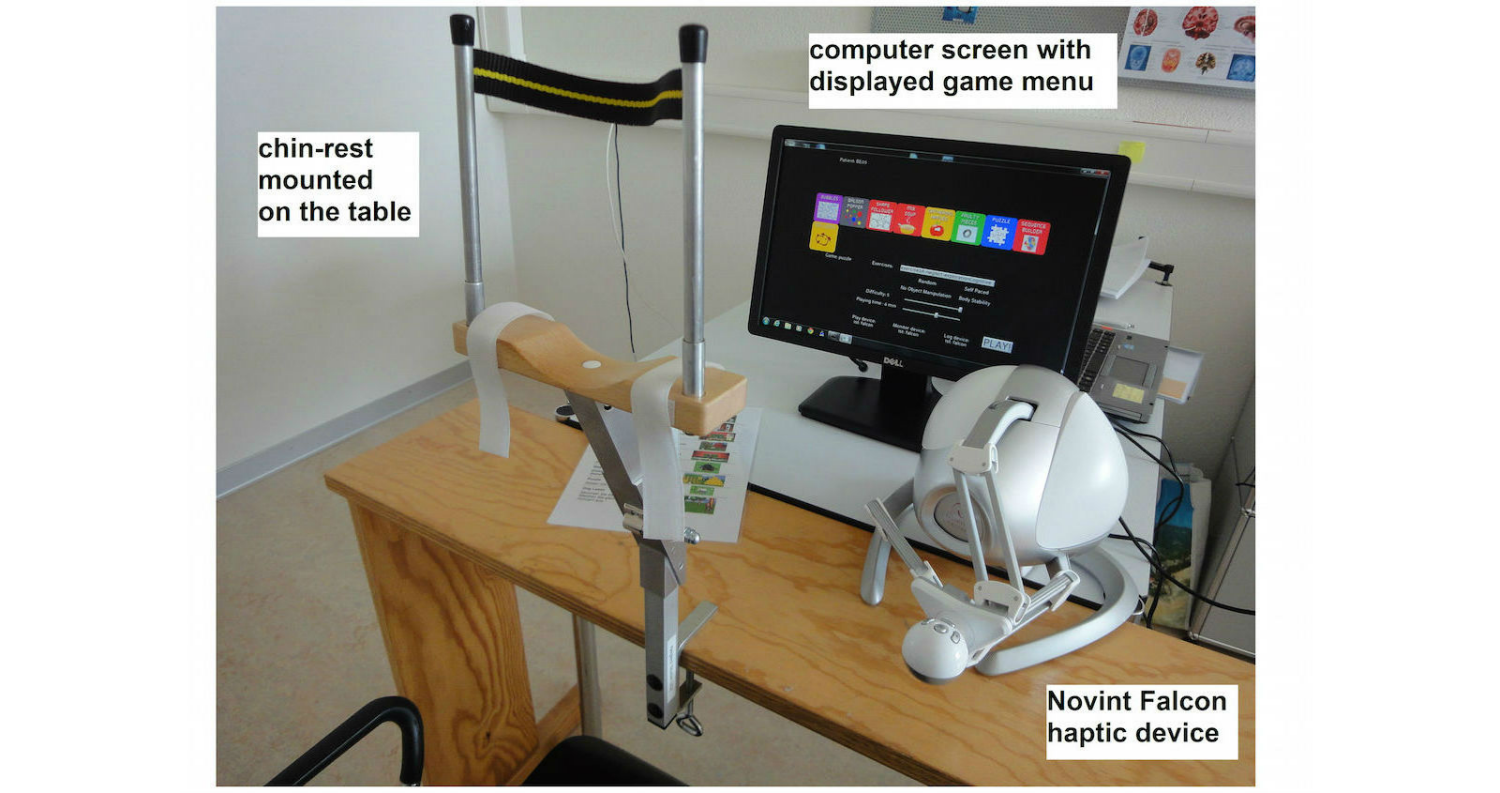
Setup of the exergames training station.

Patients were seated at a table in front of a 21-inch computer monitor at a distance of 60 to 65 cm in order to provide optimal eye tracking (Figure 1). We chose a seated position to allow more patients to participate (eg, wheelchair users) and avoid exhaustion through standing in an upright position. A height-adjustable chin rest (Novavision GmbH) was mounted on the table to avoid compensatory head movements while playing the exergames. Instead of a mouse to control games, a haptic Falcon Novint device (Novint Technologies) was used. This enabled individuals to experience a realistic sense of touch by providing simulated sensory feedback when reaching for and grasping virtual objects [62]. The Falcon Novint device can be handled with one hand only, allowing stroke patients to play the exergames with their nonaffected hand. The device was placed at the side of the computer monitor at a distance allowing ease of reach for the patients. The nonaffected upper extremity was positioned in approximately 45° shoulder abduction, 70° to 90° elbow flexion, and the forearm fully pronated. All participants were expected to independently complete 15 training sessions while being monitored by a supervising therapist. The supervision included observation of the patient during the intervention giving assistance where appropriate (eg, using the menu to start a new game). Observation was necessary for assistance if potential software difficulties occurred and for safety reasons for the patient, the latter being a regulation of the collaborating clinics.

### Exergames

The intervention program consisted of a series of 9 exergames performed while seated. The games were designed to simulate real-world tasks, such as cooking from a recipe, going for a walk with a dog, or doing a puzzle (for detailed game information on 4 games, see Pirovano et al [51]). The Falcon Novint represented, for example, a dog leash by simulating a pull from the dog to the left or right side of a virtual walking path. The increase in difficulty of all games during the training course was accomplished according to Gentile’s taxonomy of motor skills [56]. Using this systematic classification to design the exergames allowed us to design a theory-based rehabilitation program that followed the principles of exercise training (see Hoffman [53] and Ammann et al [63] for detailed descriptions of these principles). Playing time per game was adjusted from 1 to 10 minutes per game depending on the patient’s ability to concentrate playing the VR game while maintaining a seated position. After initial training and instructions were given by the research team, all subsequent game adjustments during the intervention were performed by clinic staff (occupational therapists and neuropsychologists) in accordance with the patient’s wishes.

### Intervention Protocol

The VR-based VSN training intervention took place in the 2 collaborating rehabilitation clinics serving as an additional therapy option to the standard program, which comprised daily occupational, physical, and neuropsychological therapy. Each patient was asked to attend 5 30- to 45-minute sessions per week for 3 weeks. The supervising therapist individually adjusted the intensity of playing the exergames by changing the difficulty level or game duration in the game menu and by deciding if short breaks between each game would be necessary or not. In accordance with the training principle of individuality [53], which states that people respond differently to the same training stimulus, the patient selected 3 to 4 REWIRE VSN exergames from the game menu to be played in each session. The choice was based on personal interest of the patient, which was assumed to enhance motivation while playing. During the 3-week intervention time, patients were allowed to change games if they wanted to test another one or felt bored with the previously played game. After a break of 4 weeks, a follow-up measure was performed aiming to test the training principle of reversibility, which states that the ability to maintain performance is reduced when the training stimulus (the exergames) is removed.

### Assessments

#### Primary Outcome

In order to measure the likelihood and extent to which our intervention can be fully implemented as planned and proposed [57], we designed a training diary as a protocol to document attrition, adherence, and safety issues. This training diary was on hand in the collaborating clinics and completed after each training session by the clinic staff in presence of the participating patient. The type of games played including difficulty level according to the Gentile’s taxonomy, effective training time, and patient subjective statements regarding their perceived health condition after training (posed question: “How do you feel after training: fit or tired?”) were all noted in the training protocol. Additionally, any adverse events related to the exergames intervention were noted. Potential adverse events could have been a recurrent stroke or other medical emergencies due to the early stage of recovery or an epileptic seizure or cybersickness due to playing the exergames [64]. For attrition, the number of participants lost during the intervention was registered. For adherence, participant engagement with the intervention was noted. We expected a good adherence to the intervention, defined as an attendance of at least 50% of the maximum 15 possible training sessions. Adherence was then calculated as the number of completed training sessions as a percentage of the maximum 15 possible training sessions.

#### Secondary Outcomes

##### Overview

In order to test limited efficacy of our intervention [57], the Eye Tracker Neglect Test (ETNT), Zürich Maxi Mental Status Inventory (ZüMAX), and Neglect Test (NET) were administered by the research staff at baseline and after the intervention. After a 1-month follow-up, the ZüMAX and NET were repeated either in one of the collaborating clinics or at the patient’s (new) residence (home or retirement home), depending on the length of rehabilitation stay.

**Figure 2:**
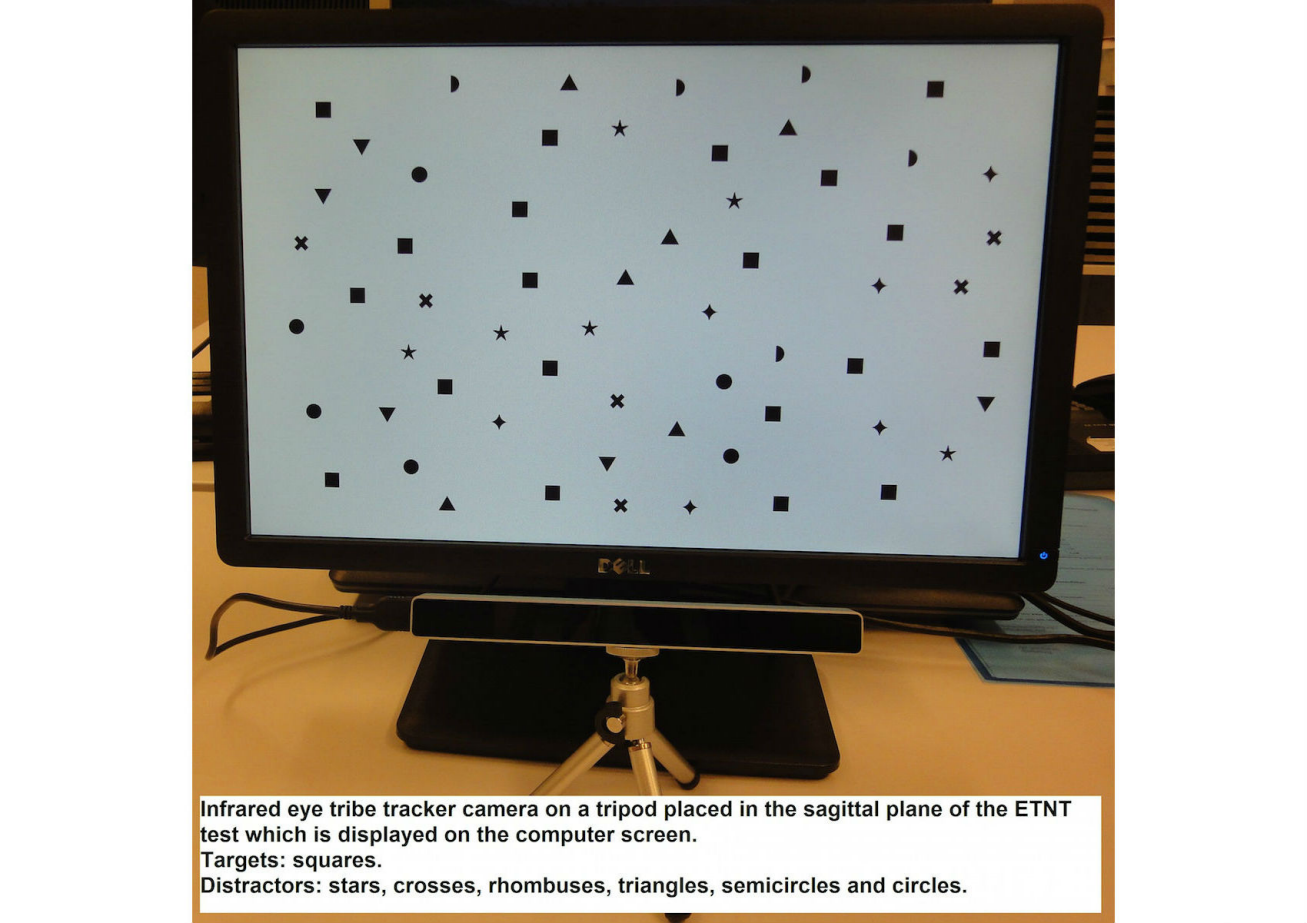
Setup of the Eye Tracker Neglect Test.

##### Spatial Exploration Skills

The ETNT is an adapted version of the cancellation test developed by Rabuffetti et al [34], aiming to assess symptoms of neglect. In contrast to the original test, in which targets are tagged by finger touch as measured by a touch screen, target detection is operated by eye fixation as measured by an eye tracker (Figure 2). The ETNT display consists of a uniform distribution of 60 stimuli including 20 targets (squares) and 40 distractors (other than square shapes) divided equally into 30 stimuli (10 targets, 20 distractors) on the right and left sides of the computer screen. The eye point of gaze was tracked with the Eye Tribe Tracker camera (Eye Tribe). Eye tracking is the process of using sensors to locate features of the eyes and estimate where someone is looking [65]. The technology relies on infrared illumination so that it does not interfere with the visual scenario. Since the system tracks eye movements relative to the sensor/screen, it is necessary to fix the head position, since head movements would be wrongly assumed as eye movements. Therefore, the patient’s head was fixed on a chin rest. In our study, each participant was seated in front of the screen with the midsagittal plane of the trunk aligned with the center of the screen. An initial calibration of the Eye Tribe Tracker camera was then followed by 1 test trial with only 4 targets and 8 distractors and the ETNT with 60 stimuli. A stimulus was counted as being found (being circled) if the patient maintained his or her point of gaze for at least 0.4 seconds within an area surrounding the stimulus with a diameter of 7% of the total screen width, in keeping with Blignaut et al [66]. There was no time constraint; patients were instructed to inform the researcher when they had finished the test. This procedure was chosen to impose no stress on the patient while exploring the targets on the screen. However, if the patient got lost or became tired, the researcher present during the test asked the patient if he or she had the impression of having found all targets and then stopped the test depending on the patient’s response.

**Figure 3:**
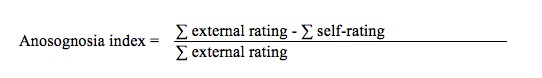
Formula for anosognosia index.

The Neglect Test (NET) consists of 7 paper-and-pencil subtests (letter and star cancellation, copying 3 figures, and line crossing and bisection with a total possible score of 70 points) and 10 behavioral subtests (representational drawing, scanning 3 pictures, menu and article reading, telling time, setting time on a digital and analog watch, and address copying with a total possible score of 100 points) designed to identify a wide variety of visual neglect behaviors [67]. It has been shown to be a robust predictor of VSN and is a predictor of functionality after stroke [68]. To assess the level of anosognosia for VSN after stroke, self-ratings of performance in 6 subtests of the NET (figure copying, star cancellation, line crossing and bisection, representational drawing, and article reading) were contrasted with external performance ratings of the examiner on a 5-point Likert scale (ranging from 1=severe difficulties to 5=no difficulties). The degree of unawareness for VSN was quantified as proposed by Vossel et al [69] (see Figure 3).

This anosognosia index (AI) will be smaller than 0 if the patient suffers from anosognosia, indicating an overestimated self-performance to what objectively has been performed. If the patient is able to rate his or her performance realistically, thus being below or matching the external rating, the index becomes equal to or greater than 0, indicating no signs of anosognosia.

##### Cognitive Skills

The ZüMAX is a domain-specific assessment tool measuring cognitive impairment by evaluating executive function, language, praxia, visual perception and construction, and learning and memory (see Tobler-Ammann et al [70] for a detailed description of the test). Each of the 5 domains allows a maximum score of 6 points, with a maximum possible test score of 30 points, representing optimal cognitive functioning. The ZüMAX has moderate to good test-retest reliability for the total test scores in patients 6 months or more poststroke and may discriminate between this patient group and healthy age and gender matched persons [70]. The ZüMAX visual perception and construction domain is the one indicating VSN symptoms. The task for visual perception is to recognize and name degraded figures, unfamiliar scenes, and a face. The task for visual construction consists of copying a figure. This assessment was chosen due to its advantage of providing both general information about poststroke cognitive impairment and neglect-specific information and because of its origin in Switzerland where the study took place and, therefore, matching the cultural background of the participants.

### Data Analysis

Data analysis was carried out using SPSS for Windows version 23.0 (IBM Corp). A Shapiro-Wilk test was administered and quantile-quantile plots were drawn to test normality of the data. The results confirmed our assumption of nonnormally distributed data due to the small sample size (*P* ≤.05 for most parameters). We therefore used nonparametric tests for data analysis. Accordingly, a Wilcoxon signed rank test was adopted to compare post- with preintervention results and follow-up with postintervention results. The Friedman test was used to test for differences in the NET and ZüMAX between all 3 measurement time points [71]. We analyzed the data for each individual and for the whole group. The fact that the ZüMAX and NET comprise subtests and the NET additionally provides a conversion table to transform raw scores into standard scores allowed us to use the Wilcoxon signed rank test not only on a group level but also on an individual level. For calculations per patient, we used the achieved standard scores of each subtest as variables, resulting in 17 variables (corresponding to the 17 NET subtests) for the NET and 5 variables (corresponding to the 5 ZüMAX domains) for the ZüMAX. For the analysis on a group level, we compared the achieved total scores per measurement point of the 7 patients.

The ETNT data were provided by the software described in Rabuffetti and colleagues [34]. A subset of the relevant indexes—namely those that were related to visual perception—was used for data analysis as only these items were suitable for the adapted test version. As the ETNT software provided 1 value per index and patient, we used the Wilcoxon signed rank test to analyze post- to preintervention changes within the sample. Additionally, we graphically displayed the individual changes post-pre intervention by drawing the performed search path and fixation points and creating heat maps to visualize group changes post-pre intervention.

**Figure 4:**
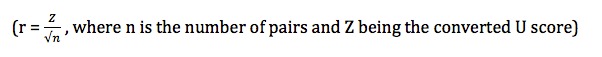
Cohen formula for nonparametric tests.

In order to perform an a priori power analysis to determine the minimum sample size for a future randomized controlled trial, we calculated the effect sizes for the secondary outcome measures. We applied the Cohen formula for nonparametric tests [72] (see Figure 4). Accordingly, small, medium, and large effect sizes were labeled as *r*=0.1, 0.3, and 0.5, respectively [73]. The level of significance was set at *P* ≤.05.

## Results

### Overview

From the 18 VSN patients consecutively screened for eligibility in both clinics from March 2015 to March 2016, 7 patients (39%) were eligible and consented to participate in this study, therefore taking part in the VR exergaming program including baseline, postintervention, and 3-month follow-up measures. Reasons preventing patients from participating were suffering from a right-sided VSN due to a left brain lesion, having a severe apraxia (fewer than 5 points on the TULIA (AST) screening instrument, and being in a poor health condition confining them to bed. Patient characteristics are presented in Table 1.

**Table 1:** Patient characteristics.

Patient		Age, years	Sex	Days post-stroke at study entry	RBL^a^ stroke type	Handedness/ affected hand (function)	Education	Locomotion	CBS^b,i^	AST^c,j^
P1		64	M	25	ischemia	R/L (none)	PE^d^	WC^e^	7	12
P2		74	M	29	ischemia	R/L (back)	PE	W^f^	17	12
P3		64	M	114	ischemia	R/L (none)	PE	WC	5	12
P4		70	M	32	ischemia	R/L (back)	SE^g^	WC	6	12
P5		53	M	42	ischemia	R/L (none)	PE	W	5	12
P6		78	F	35	hemorrhage	R/L (back)	SE	W	10	12
P7		77	F	47	hemorrhage	R/L (back)	PE	WC	16	9
**IQR^h^**										
	25	64	—	29	—	—	—	—	5	12
	50	70	—	35	—	—	—	—	7	12
	75	77	—	47	—	—	—	—	16	12

^a^RBL: right brain lesion.

^b^CBS: Catherine-Bergego Scale.

^c^AST: Apraxia Screen of TULIA.

^d^PE: primary education.

^e^WC: wheelchair.

^f^W: walker.

^g^SE: secondary education.

^h^IQR: interquartile range.

^i^Maximum score = 30 (severe neglect); 0 points = no neglect.

^j^Maximum score = 12 (no apraxia); threshold for apraxia: ˂9 points; severe apraxia: ˂5 points.

### Primary Outcome

An overview of individual (P1-P7) and group (interquartile range [IQR], mean) results in the training protocol is shown in Multimedia Appendix 1. There were no adverse events and drop-outs during the intervention. A median attendance of 14 (IQR 12-15) training sessions (maximum 15 sessions) was achieved, which corresponds to a median adherence of 93% (IQR 80%-100%). Reasons for nonparticipation were of organizational or medical nature (eg, overlap with other therapy sessions or due to fatigue) rather than because of motivational factors. All patients played 2 to 4 games and repeated at least 1 game per training session. The supervising therapists adapted and individually progressed the patient training protocols on a weekly basis during the exergames intervention in accordance with patient progress. If, for example, the patient got bored with the current difficulty level of the played game or the therapist observed that the game was played without effort, the therapist modified the difficulty level within each game. However, if the patient had reached the most difficult level, the therapist replaced easy games with more complex ones (ie, games including more distractors or moving objects). An analysis of the progress as measured by the achieved game scores was therefore not feasible, as progression in difficulty resulted in a temporary decrease in game scores. Instead, the progress in the exergames training of the 7 individual patients was documented weekly according to the Gentile’s taxonomy of motor skills. These results are shown in Multimedia Appendix 1.

### Secondary Outcomes

#### Spatial Exploration and Cognitive Skills

**Figure 5:**
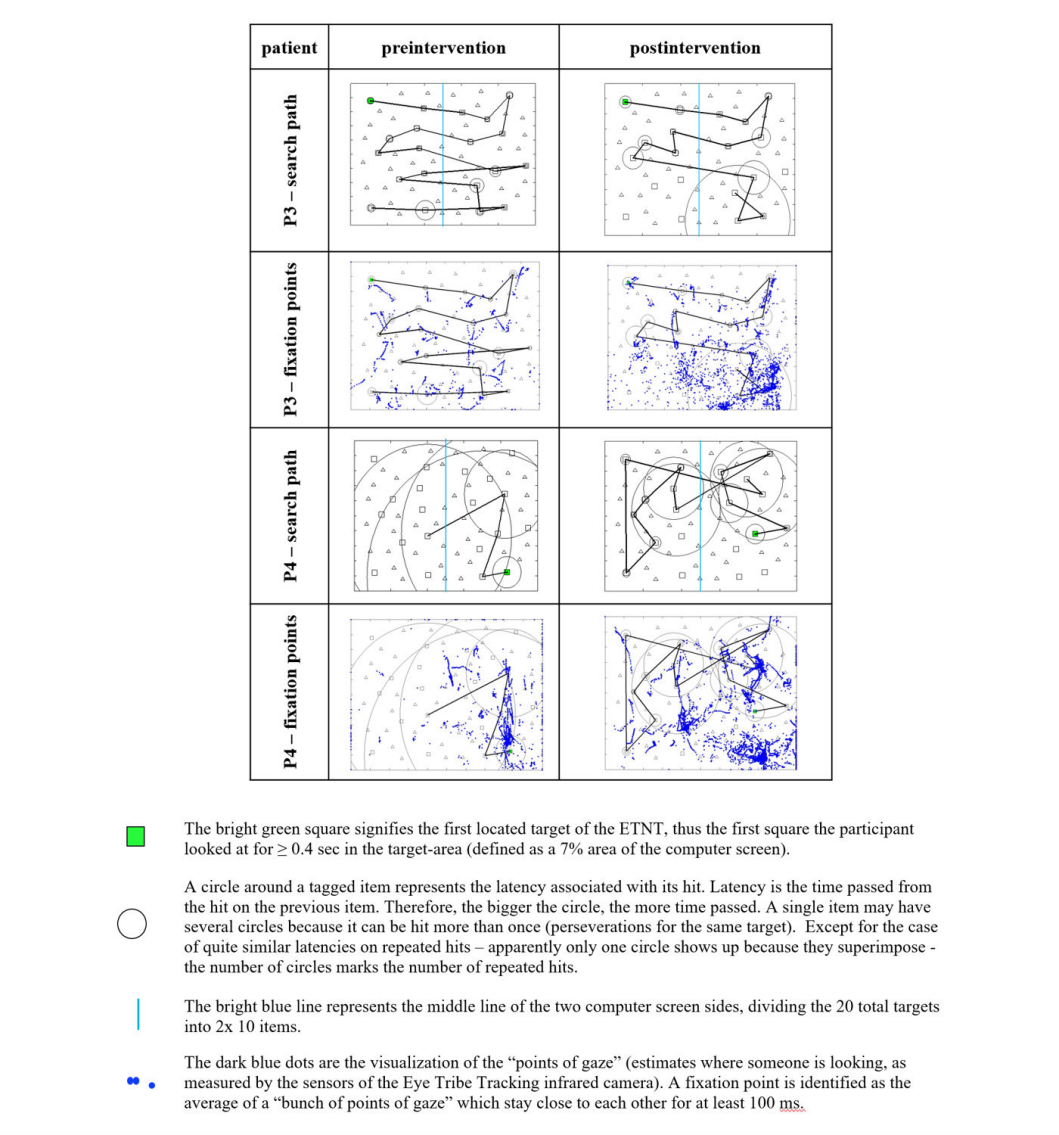
Examples of pre- and postintervention results of the Eye Tracker Neglect Test search paths and fixation points of P3 and P4.

**Figure 6:**
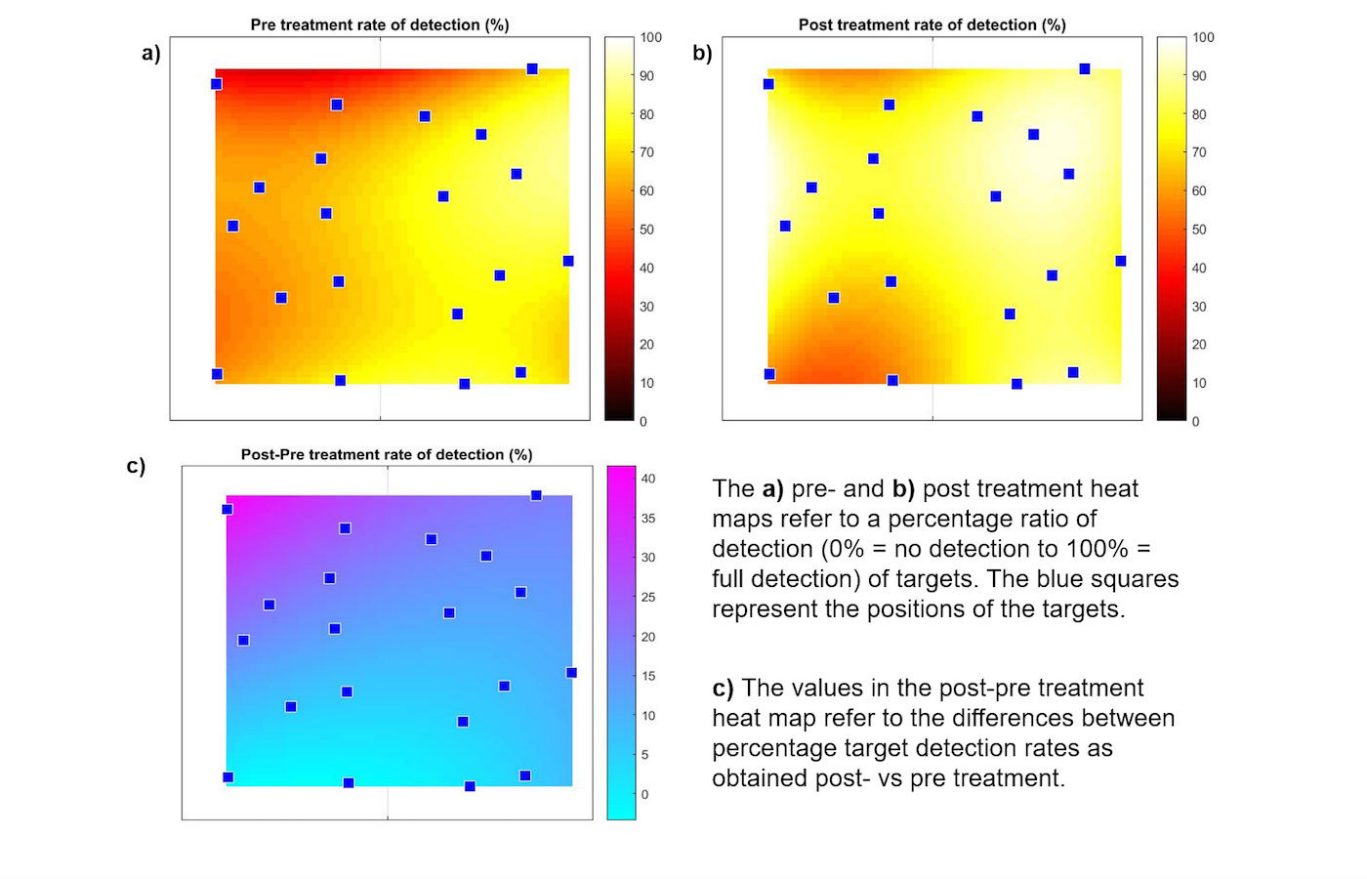
Heat maps of the preintervention, postintervention, and difference post-pre results of the Eye Tracker Neglect Test.

**Figure 7:**
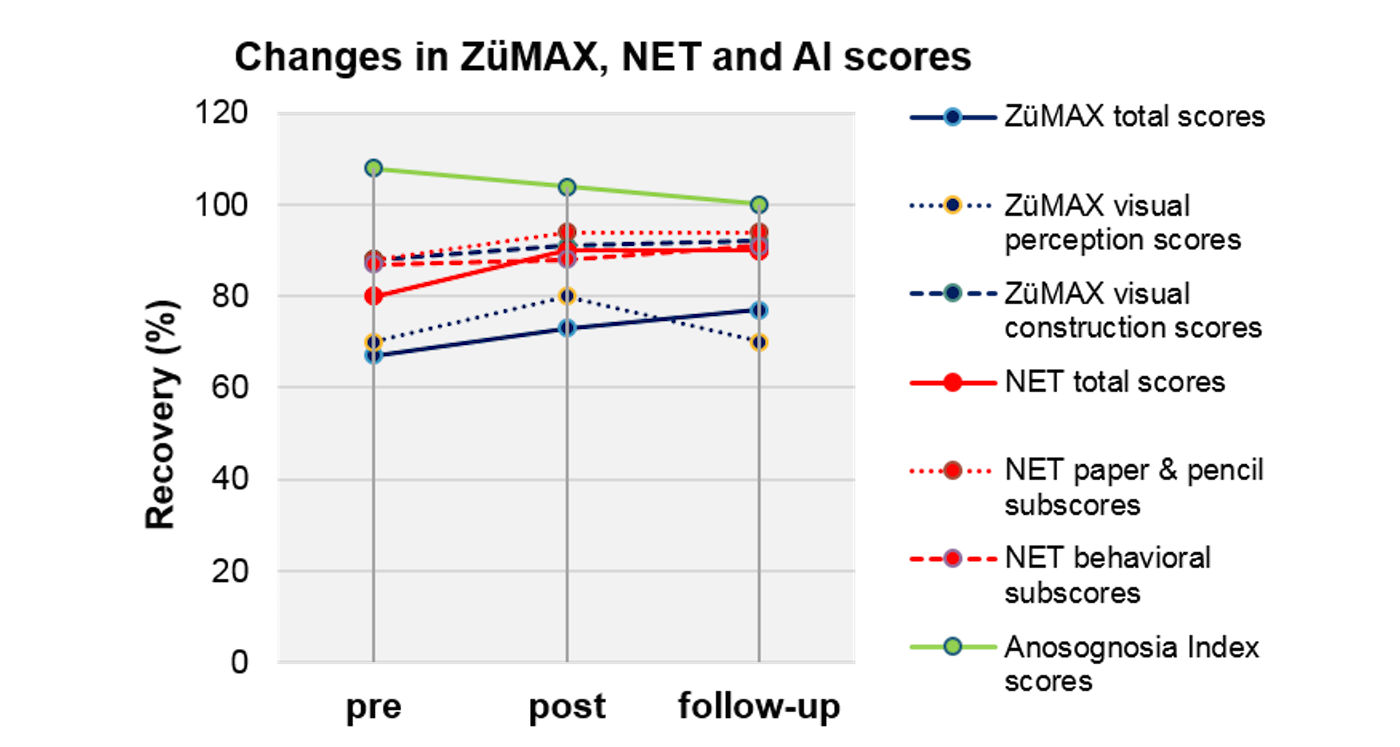
Graphical overview of preintervention, postintervention, and follow-up Zürich Maxi Mental Status Inventory, Neglect Test, and anosognosia index results.

An overview of the individual ETNT scores and group changes post- to preintervention is shown in Multimedia Appendix 2. Figure 5 shows 2 examples of pre-post intervention search path strategies and fixation points as measured by the eye tracker camera (see Multimedia Appendices 3 and 4 for all graphs of individual post-pre ETNT search paths and fixation points). Figure 6 shows the heat maps of the pre- and postintervention and differences in post-pre detected targets of the ETNT. An overview of ZüMAX, NET, and AI scores and group changes pre-, postintervention, and at follow-up is summarized in Multimedia Appendix 5 and graphically displayed in Figure 7 (overview) and Multimedia Appendix 6 (individual results per outcome measurement). The individual changes in the ZüMAX and NET assessments are presented in Multimedia Appendix 7.

#### Eye Tracker Neglect Test

##### Group Level

The pre-post assessment showed a median group trend of slight improvement in the total located targets from 15 to 16 (+6%) pre-post, which was concomitant with an increasing median total test duration (+33.9 seconds pre-post) (Multimedia Appendix 2). The postintervention performance showed a median decline of 2 (IQR 0-4) missed targets on the left side of the screen and a median increase of 1 (IQR 0-3) missed target on the right side of the screen. The heat maps (Figure 6) indicate that the ability to detect targets in the upper left portion of the computer screen increased postintervention and remained substantially unmodified otherwise. The results further showed an unchanged median group trend pre-post intervention regarding the neglect score, the median latency, and proximity. There were no statistically significant changes pre-post intervention for any ETNT parameters (Multimedia Appendix 2).

##### Individual Level

P4 improved from 5 to 15 total located targets pre-post intervention (Multimedia Appendix 2 and Figure 5). The only individual worsening pre-post regarding the left spatial exploration skill was P3, with no target missed preintervention and 4 targets missed postintervention (Figure 5). Most participants (6/7, 86%) started their search in the right sector and scanned leftwards vertically (Multimedia Appendices 3 and 4), while P3 started in the upper left sector and scanned top-down horizontally (Figure 5). Additionally, 4 out of 7 (57%) started their search on the right side of the screen, 2 out of 7 (29%) were able to change the starting point from right to left, and 1 participant (P3) started the search on the left side of the screen pre- and postintervention (Multimedia Appendices 3 and 4).

#### Neglect Test

##### Group Level

The NET showed statistically significant improvements pre- to postintervention (*P*=.01 to *P*=.03) in the total score and both subtests as well as in the Friedman test (*P*=.01 to *P*=.02) (Multimedia Appendix 5). The AI results showed a group trend toward zero (median 0.08 pre, 0.04 post, 0 follow-up), indicating a trend toward perfect awareness of oneself (Multimedia Appendix 5).

##### Individual Level

P1 and P2 declined at follow-up in the NET total scores, while P3 to P7 showed further improvement. P1, P5, and P7 significantly changed in their NET total scores, P1 post–follow-up (*P*=.04) and P5 and P7 pre-post–follow-up (Friedman test; *P*=.01 and *P*<.001, respectively) (Multimedia Appendix 7). The post–follow-up result of P1 also showed a large effect (*r*=–.80), indicating a large decline in the NET scores postintervention, especially in the paper-and-pencil subtasks (Multimedia Appendix 6). Analyzing NET scores by subtests, all patients improved pre- and postintervention in the paper-and-pencil subtests (Figure 7), while in the behavioral subtests, all patients improved pre- to postintervention except for P3 who remained unchanged (Multimedia Appendix 6). Two patients (P3 and P7) suffered from anosognosia with a trend of aggravation from pre- to follow-up assessments (Figure 7). P1, P2, and P4 showed no signs of anosognosia at follow-up (AI index=0), while P5 and P6 showed a trend toward an increasing AI index, underestimating their actual performance (Multimedia Appendix 6).

#### Zürich Maxi Mental Status Inventory

##### Group Level

The ZüMAX showed improvements in the total scores from pre- to postintervention to follow-up (Figure 7) that were not statistically significant (*P*=.29 to *P*=.45, median +2 points pre-post, +1 point post–follow-up) (Multimedia Appendix 5).

##### Individual Level

On an individual level, P1, 2, 5, and 6 improved from pre- to postintervention in the ZüMAX total scores, while P3 and 4 declined and P7 remained unchanged (Multimedia Appendix 6). The post–follow-up scores declined in P1 and P7, while P2 to P6 still improved their post–follow-up scores, with P3 and P4 declining pre-post intervention. In the ZüMAX visual perception subtask (recognizing and naming degraded figures), 3 of the 7 patients stayed unchanged from pre-post to follow-up (P3, P5, P6), while the largest progression was apparent in P7, with a clear decline of 3 points after termination of the exergames intervention (Multimedia Appendix 6). In the second ZüMAX subtask related to neglect (visual construction: figure copying), 3 patients performed the pre- and postintervention assessments with unchanged scores (P3, P6, P7), and 3 patients showed a slight decline post–follow-up (P1, P3, P5).

## Discussion

### Principal Findings

This study evaluated the feasibility of an exergames intervention aimed to affect VSN symptoms in patients early poststroke in terms of implementation (adherence, attrition, and safety) and limited efficacy testing by documenting changes in VSN symptoms. The exergames intervention was tolerated well by all participants and was mainly performed without major difficulties, showing that its implementation in the clinical setting was feasible. With 0 out of 7 (0%) attrition, no adverse events, and a median adherence rate of 14 out of 15 sessions (93%), the compliance of the patients to the exergames was excellent. Such a result was possibly due to the clinic staff’s commitment, as the VR intervention was smoothly integrated into the daily therapy schedule of the clinic. However, as we aimed to test the fit of our intervention in a real-world setting, we prioritized clinic constraints over optimal conditions and settings. As a consequence, this priority reduced potential omissions of training sessions as described in purely home-based VR interventions [46]. There, the level of use of the VR system was variable and fell far short of recommendations, despite the weekly or biweekly visits of a researcher to the patients’ homes to check progress and retrieve data.

Other studies testing novel VR systems for upper limb stroke rehabilitation have also shown high levels of adherence to the training intervention [74-76]. However, these patients were in the chronic stage of recovery and did not suffer from USN. The single participant with USN of the Duckneglect study (Mainetti et al [41]), who also was in the chronic stage of stroke recovery, showed an excellent adherence to the exergames, in keeping with our case series.

Regarding the exergames training, the median duration per session was 30 (IQR 23-30) minutes, which fell short of the planned 30 to 45 minutes of training time. For our study, we decided to set a timeframe rather than an exact exposure time, because little is known about the optimum duration and patterning of training exposure to virtual environments [77]. Possible reasons for the rather short training sessions in our sample were twofold: either patients (eg, P2 and P3) were quite fit and finished the planned exergames session early or, conversely, were too tired to play longer (eg, P1). In our sample, 5 out of 7 (71%) patients indicated being fit after most trainings (median 13 out of 15 [87%] possible sessions), excluding fatigue as being a main reason for the rather short training time. P7, however, being the only participant with a highly distinctive VSN as measured with the NET, needed on average 34 minutes to accomplish the games. These results highlight the importance of adjustability of the difficulty level of the exergames to the functional ability of the stroke patient [76], thus avoiding a decline in enjoyment level while playing [77]. Given the uncertainty about optimal exposure together with our small and highly varied sample (Table 1, CBS scores), future trials should focus on investigating optimal thresholds of exposure time corresponding to the degree of VSN severity.

The fact that there were no adverse events during the training period in our case series was encouraging. Besides being lucky that no recurrent stroke or other medical emergency happened during the intervention, the design of the games might have contributed as well to the safety of our participants. For example, implementing both stationary and in-motion conditions of the virtual scenario together with the option of choosing between intertrial variability and no intertrial variability while gaming allowed the patient to choose the optimal virtual environment to be challenged on the one hand but not be overwhelmed on the other hand (see Multimedia Appendix 1). Allocating these options in difficulty level might have contributed to reducing the risk of cybersickness while playing despite the stationary condition of the patient [64]. When designing the setup of our study, we intentionally planned a seated position to play the games. This allowed patients to fully concentrate on the exploration of the neglected space without having to invest energy standing in an upright position. Furthermore, playing the games in a seated position contributed to the prevention of falls. Prahm et al [78], for example, also designed a game-based intervention in a seated position reporting no adverse events. However, their participants were able-bodied adults. Wiloth et al [79] reported no adverse events in their game-based assessment to measure motor-cognitive function in people with dementia while they were standing on a movable platform. Although falls are highly prevalent in people with cognitive impairment such as dementia [80], people poststroke additionally suffer from motor impairment. Despite their hemiparesis, our participants were all able to perform the games and handle the Falcon Novint with the nonaffected hand. The clinic staff reported that sometimes the more concentrated or—toward the end of the training session—the more tired the participants became while playing the exergames, the more they tilted to the left side with their upper body. As this is a well-known phenomenon in VSN patients poststroke [81], we think our approach of offering gameplay in a seated position guaranteed patient safety. The rather tilted position, however, did not prevent the participants from continuing to play the exergames.

Our limited efficacy testing showed a group trend of improvements in cognitive and spatial exploration skills. However, these changes cannot be exclusively attributed to the exergames intervention. One reason is the ongoing VSN treatment in the rehabilitation clinic that might also explain some of the improvements. A further possible reason is spontaneous recovery of VSN symptoms not only during the acute phase after stroke but also during the following few weeks. Paolucci et al [82], for example, reported a decrease of VSN symptoms to 20% from 45% after 1 month poststroke, which may also have occurred in our sample. Additionally, the heterogeneity in our sample regarding neglect severity—4 out of 7 (57%) were only mildly affected with CBS scores between 5 to 7 points (Table 1), while 2 out of 7 (29%) were severely affected (16-17 scores on the CBS)—might have influenced the rate of improvements, too. However, our sample did not show a ceiling effect—being present if 15% or more participants achieve the highest possible score [83]—as no participant achieved the highest score in any of the outcome measurements. Furthermore, most patients continued improving their scores in the NET and ZüMAX assessments postintervention, achieving the highest scores after a break of 4 weeks (follow-up). Only P1 in both tests and P2 (NET) and P7 (ZüMAX) showed a decline in scores from post to follow-up, which would correspond to the training principle of reversibility [53,63] that states that once a training stimulus is removed, performance levels will eventually return to or below baseline. Comparing our efficacy testing results with literature was difficult, as studies with a similar setup, time point of measurement, and target group are scarce. There is evidence that training with VR methods improve spatial attention and show transfer of improved spatial attention in activities of daily living in chronic neglect [30,32]. Kim et al [84] showed additional benefit for treating cognitive impairment in stroke patients without VSN in the subacute phase of recovery when adding VR training to classical cognitive rehabilitation. The evidence supports our findings that using VR systems to treat cognition in stroke patients is promising and feasible; however, further research is warranted and necessary to test its use in patients with VSN symptoms early poststroke. Future studies with a focus on treatment effects using controlled research designs should be used to assess causal relationships between the game-based interventions and important patient outcomes.

The median AI values in our sample ranged from 0.08 preintervention to 0.0 at follow-up. Comparing those results to bigger RBL stroke samples with USN—mean (SD) lower AI –0.28 (0.5) for n=34 and mean (SD) higher AI –0.47 (0.5) for n=22; Vossel et al [69] and mean (SD) AI –0.16 (0.38) for n=55; Vossel et al [6]—our 7 patients showed quite a high level of self-awareness for their visuospatial deficits, including P3 and P7 who scored below zero (indicating anosognosia) during all 3 measurements. Of the 7 patients, 3 continued increasing their level of self-awareness postintervention to zero (P1, P2, P4), suggesting positive effects of time poststroke on anosognosia rather than our training intervention. However, time poststroke might not be a viable indicator for those continued improvements in self-awareness of neglect, as Vossel and colleagues [6] found no differences in their AI values across their 3 patient subgroups differing in time since stroke onset. Therefore, continued rehabilitation might be a plausible reason for those further improvements. However, looking at the other 4 patients, AI values showed a tendency toward worsening from preintervention to follow-up, with P3 and P7 overestimating and P5 and P6 underestimating their NET performance, although they also had continued rehabilitation. There is evidence that anosognosia for spatial deficits is not predominant, with different tasks evoking different degrees of awareness about the neglect symptoms [85]. As the AI is calculated on the basis of 4 paper-and-pencil and 2 behavioral tasks of the NET, it might be that this mix of tasks also influenced the miscellaneous AI results. For example, Ronchi et al [85] found that anosognosia level improved after performance of complex visuomotor (eg, cancellation and drawing) and reading tests. By contrast, the self-rating in line bisection tasks was not related to actual task performance [85]. Furthermore, as the AI test is designed to be performed after task execution, it might be that the patients were able to correct their erroneous self-rating to some extent at least in the complex visuomotor tasks. Last but not least, the repetition of the NET test over a relatively short time span might have influenced the AI results, too, as the patients knew that a self-rating would follow after certain tasks.

The ETNT results are to be considered preliminary and should be interpreted with caution. Calibration difficulties with the Eye Tribe Tracker system (eg, most patients only reached 3 out of 5 points in calibration quality scores) may have influenced the reliability of the setup and the accuracy of the results. The calibration consisted of eye-tracking a circle that moved around the whole display. The difficulties experienced by patients in following the rapidly moving circle and the requirement to look at calibration points at the very left of the computer screen were the main reasons for the rather poor calibration results. Such difficulties may produce visible effects (in Figure 5, for example, P4 evidences a dense cloud of gaze points on the bottom right corner during the postintervention assessment, where P4 unsuccessfully tried to tag the 3 targets he could point at with his hand). Baheux et al [86] also reported calibration problems with their 3-D haptic VR system coupled with an eye-tracking device. They assumed that the VSN patient spectacle wear or eye color might have been reasons for calibration difficulties. However, these calibration difficulties notwithstanding, our ETNT results showed trends toward slight improvements in both total located and missed targets on the left side of the screen. The heat maps display the increased ability to detect targets in the upper left portion of the computer screen postintervention but remained substantially unmodified otherwise. However, the preintervention performance was already fairly good in our sample. Furthermore, the heat maps show that the ETNT can identify neglected areas. This is in line with the Rehabilitation Gaming System by Maier et al [31] using the Kinect motion capture system being equally able to measure symptoms of neglect. In contrast to our test, the stroke patients in the chronic stage of recovery explored the neglected side with the paretic arm.

The individual search strategy (Multimedia Appendices 3 and 4) of most patients was comparable with those described by Müri et al [65] and Rabuffetti et al [34], namely to start in the (extreme) right sector and scan leftwards vertically. This contrasts with the search strategy demonstrated by the control subjects in these studies, which started in the upper left sector and scanned top-down horizontally (like reading). Only P3 showed a nonneglect-specific search strategy (Figure 5). The 4 missed targets in the left lower corner postintervention, which P3 was able to detect preintervention, were due to calibration difficulties, as P3 was able to point at those 4 targets with his hand. P1 and P6 were the only patients able to shift their search starting points from the right to the left side from pre- to postintervention, indicating improvements in exploring the neglected side [34]. Not surprisingly, the increased total test duration pre- to postintervention in our sample went along with slightly more detected targets. When the participant increases the number of detected targets, it can be the case that previously neglected targets are now detected albeit with a fairly large latency. We interpret this as positive since more visual space is actively explored but latency and concomitantly the total test duration may therefore also increase. A future study including a larger sample and a control condition is, however, warranted to substantiate or refute these findings.

We performed an a priori power analysis to determine the minimum sample size for such a future trial [87]. Specifically, we assessed the requirements for a randomized controlled study with an experimental group (receiving exergame-based therapy and usual stroke rehabilitation) and a control group (receiving usual stroke rehabilitation only). Assuming an effect size of *r*=0.9 (based on our observed value for NET total scores, Multimedia Appendix 5), acceptable type I and II error probabilities (0.05 and 0.20, respectively) may be obtained with a minimum sample of 34 subjects per group for a 2-group pre-post-test design. To account for attrition, initial sample size should be increased to 45 subjects per group [88]. Given the fact that only 18 potential participants were available within 12 months of recruitment, we recommend collaborating with more than 2 clinics for such a trial.

### Limitations and Future Work

The length of the training phase was rather short (ie, 3 weeks). We deliberately did not choose a longer training period, as we primarily wanted to test the exergames’ feasibility and not their effect on VSN symptoms. On the other hand, the rather short training phase allowed us to keep the drop-out risk relatively low (eg, due to discharge home during the training phase). In a next step, it would be important to test the exergame system’s feasibility in patient homes to evaluate adherence, safety, and attrition to using the system in this setting, as the provision of novel home-based rehabilitation options was the main goal of REWIRE. In this setting, a longer training phase could be tested. Furthermore, a progression as measured by the game scores should be implemented together with an immediate graphical feedback after each training session to enhance motivation for playing the exergames. For this implementation, ideas from the rehabilitation method of shaping [54], where frequent feedback and encouragement during training are central, could be adopted. In order to maximize confidence that changes in outcomes can be attributed causally to the exergames intervention, a control group in a pilot randomized controlled trial design would be needed. The neglect exergames should further be designed to switch levels of difficulty (ie, progressing from the right to the left side of the screen or vice-versa). Designing this option would allow recruiting stroke patients with a left-sided brain lesion and VSN symptoms, too. By excluding them in our project we were aware that we would probably miss some patients having ipsilesional neglect [89], which would have made a participation in the exergames intervention feasible. However, as left-sided neglect is quite rare compared to right-sided neglect [5], the risk of missing such an ipsilesional neglect patient was relatively low.

The ETNT could further be developed regarding the following:

- Calibration procedure of the Eye Tribe Tracker by reducing the speed of the circle to be followed, for example
- Software indexes, which were initially designed for the touchscreen (hand-eye coordination) test. Indexes important for eye-tracking would be, for example, the cumulative fixation duration, spatial distribution of fixations in the horizontal and vertical plane, or the number and amplitude of exploratory saccades as explored by Müri et al [65]
- Collection of the search strategy patterns of age-matched controls

Additionally, future work could correlate ETNT measures to scores in standardized clinical scales, such as the NET scores, in order to validate the derived ETNT measures of recovery after VSN.

### Conclusion

This study showed that patients adhered well to the REWIRE neglect exergames intervention with no drop-outs, no adverse events, and an adherence rate of 14 out of 15 sessions (93%). We therefore judged this intervention to be safe and feasible for VSN patients early poststroke and appropriate for further testing. Cognitive and spatial exploration skills, as evaluated using ETNT, NET (spatial exploration), and ZüMAX (cognition) assessments, improved in most patients from pre- to postintervention. The results of the amount of exergames use is promising for future applications and warrants further investigations, for example, in the home setting of patients as a motivating training tool to complement usual care and support augmenting training frequency and intensity in RBL stroke patients with VSN.

## Appendix

Multimedia Appendix 1 Overview of individual and group results in the training protocol.

Multimedia Appendix 2 Overview of individual Eye Tracker Neglect Test scores and group changes pre- to postintervention.

Multimedia Appendix 3 Pre- and postintervention results of individual Eye Tracker Neglect Test search paths.

Multimedia Appendix 4 Pre- and postintervention results of individual Eye Tracker Neglect Test fixation points.

Multimedia Appendix 5 Overview of Zürich Maxi Mental Status Inventory, Neglect Test, and anosognosia index scores and group changes preintervention, postintervention, and follow-up.

Multimedia Appendix 6 Graphical display of individual Zürich Maxi Mental Status Inventory, Neglect Test, and anosognosia index scores.

Multimedia Appendix 7 Individual changes in the Zürich Maxi Mental Status Inventory and Neglect Test assessments.

## Abbreviations

AI: anosognosia index
AST: Apraxia Screen of TULIA
CBS: Catherine Bergego Scale
ETNT: Eye Tracker Neglect Test
IQR: interquartile range
NET: Neglect Test
RBL: right brain lesion
REWIRE: Rehabilitative Wayout in Responsive Home Environments
TULIA: test of upper limb apraxia
USN: unilateral spatial neglect
VR: virtual reality
VSN: visuospatial neglect
ZüMAX: Zürich Maxi Mental Status Inventory
