# Supplementary material for: Exergames Encouraging Exploration of Hemineglected Space in Stroke Patients With Visuospatial Neglect: A Feasibility Study
Source: JMIR Serious Games. 2017 Aug 25;5(3):e17. doi: 10.2196/games.7923 (PMC5591404; doi:10.2196/games.7923)
Supplement: Multimedia Appendix 1 [file games_v5i3e17_app1.pdf]

training protocol parameters	attended sessions[a] n (%)	total training time (min)	mean ± SD training duration/ session[b] (min)	health condition after training n (%)		progression over the 3 week intervention[c]		
patient				fit	tired	week 1	week 2	week 3
**P1**	12 (80)	276	23 ± 3.0	2 (17)	10 (83)	2x L5 / 2A	3x L5 / 4A	3x L5 / 4A
**P2**	15 (100)	392	26 ± 4.0	13 (87)	2 (13)	3x L4 / 3A	3x L5 / 4A	4x L5 / 4A
**P3**	12 (80)	278	21 ± 2.8	10 (83)	2 (17)	4x L2 / 2A	4x L4 / 3A	4x L5 / 2B
**P4**	15 (100)	445	30 ± 1.3	14 (93)	1 (7)	3x L3 / 1A	4x L4 / 3A	3x L5 / 1A
**P5**	15 (100)	450	30 ± 0	10 (67)	5 (33)	3x L3 / 2A	3x L4 / 3A	3x L5 / 3A
**P6**	14 (93)	420	30 ± 0	14 (100)	0 (0)	3x L2 / 1A	3x L5 / 4B	3x L5 / 3A
**P7**	10 (67)	335	34 ± 10.3	0 (0)	10 (100)	3x L1 / A1	2x L2 / A2	3x L3 / B1
**IQR** 25	12 (80)	278	24.5	2 (17)	1 (7)			
**IQR** 50	14 (93)	392	30	13 (87)	2 (13)	NA	NA	NA
**IQR** 75	15 (100)	445	30	14 (93)	10 (83)			
**mean ± SD**	13.3 ± 2.0 (88.6 ± 13.2)	370.9 ± 74.8	27.7 ± 4.6	9.4 ± 5.9 (64.3 ± 39.8)	4.3 ± 4.2 (35.7 ± 39.8)	NA	NA	NA

IQR = Inter Quartile Range; min = minutes; n = number; NA = not applicable; % = percentage

[a] maximum training sessions: n = 15

[b] calculated as the total training time divided by the total number of attended sessions

[c] to be read as: 2-4x = number of exergames. L1-5: difficulty levels (the higher the more exploration needed to the neglected left side). 1A-5A or 1B-5B: Gentile's Taxonomy

progression (boxes A: no force feedback from Falcon; boxes B: force feedback from Falcon; 1A/1B: stationary conditions of virtual scenario, no intertrial variability during the game; 2A/2B: stationary conditions, intertrial variability; 3A/3B: in-motion conditions (i.e. moving virtual objects during the game, e.g. balloons), no intertrial variability; 4A/4B: in-motion conditions and intertrial variability)
