# Supplementary material for: Exergames Encouraging Exploration of Hemineglected Space in Stroke Patients With Visuospatial Neglect: A Feasibility Study
Source: JMIR Serious Games. 2017 Aug 25;5(3):e17. doi: 10.2196/games.7923 (PMC5591404; doi:10.2196/games.7923)
Supplement: Multimedia Appendix 2 [file games_v5i3e17_app2.pdf]

ETNT indexes	**total located targets**[a] (n)		**missed targets left**[b] (n)		**missed targets right**[b] (n)		**neglect score**[c] (%)		**median latency**[d] (sec) IQR 25/*50*/75		**median proximity**[e] (cm) IQR 25/*50*/75		**total test duration**[f] (sec)	
**patient**	**pre**	**post**	**pre**	**post**	**pre**	**post**	**pre**	**post**	**pre**	**post**	**pre**	**post**	**pre**	**post**
**P1**	20	20	0	0	0	0	0	0	1.3<br>*2.3*<br>3.4	0.7<br>*0.8*<br>1.0	0<br>*0*<br>1.5	0<br>*1*<br>1	71.5	20.3
**P2**	10	16	4	3	6	1	-10	10	2.3<br>*5.2*<br>23.7	7.1<br>*19.3*<br>44.1	0<br>*1*<br>5	1<br>*2*<br>4	131.7	455.6
**P3**	20	15	0	4	0	1	0	15	1.1<br>*1.3*<br>2.0	1.2<br>*2.1*<br>5.2	0<br>*1*<br>2	0<br>*0.5*<br>2.5	39.7	168.1
**P4**	5	15	9	2	6	3	15	-5	34.0<br>*49.3*<br>57.8	1.8<br>*2.8*<br>12.7	1.5<br>*2.5*<br>6.5	0<br>*1*<br>3	200.3	229.4
**P5**	20	19	0	0	0	1	0	-5	1.2<br>*2.7*<br>7.1	1.8<br>*2.1*<br>6.7	0.5<br>*2*<br>3.5	0.5<br>*1.5*<br>3	112.3	146.2
**P6**	15	20	5	0	0	0	25	0	1.8<br>*2.1*<br>6.7	1.3<br>*2.7*<br>5.7	0.5<br>*2.5*<br>4	0<br>*1*<br>3.5	114.7	97.6
**P7**	4	7	10	10	6	3	20	35	6.2<br>*6.4*<br>27.6	15.4<br>*20.7*<br>24.0	1<br>*1*<br>2.5	0<br>*0*<br>1	78.6	128.7
**IQR** 25	5	15	0	0	0	0	0	-5	2.1	2.1	1	0.5	71.5	97.6
**IQR** *50*	*15*	*16*	*4*	*2*	*0*	*1*	*0*	*0*	*2.7*	*2.7*	*1*	*1*	*112.3*	*146.2*
**IQR** 75	20	20	9	4	6	3	20	15	6.4	19.3	2.5	1.5	131.7	229.4
**R WS** *P*	.21		.27		.22		.83		.80		.31		.18	
**R WS** Z	-0.26		-1.10		-1.23		-0.21		-0.25		-1.03		-1.35	
**R WS** r	-.10		-.42		-.47		-.08		-.09		-.39		-.51	

cm = centimeters; ETNT = Eye Tracker Neglect Test; IQR = Inter Quartile Range; n = number; *P* = level of significance ($P \leq .05$); r = effect size; sec = seconds; WSR = Wilcoxon signed ranks test; Z = Z-score (approximation of the observed difference in terms of the standard normal distribution)

[a] maximum detectable targets: 20 = 100% (a target is counted if the participant looked at it for longer than 0.4 sec)
[b] maximum detectable targets: 10 on the left and 10 on the right side of the screen
[c] defined as the difference between the number of targets cancelled on the left and on the right side of the screen, expressed as a percentage of the total number of targets. 0 = no laterality to search performance; negative numbers = focus more on the left side; positive numbers = focus more on the right side
[d] summary value of all time spans between the current and the previous item hit during the test
[e] number of not yet found targets that were closer to the previous one than the actual newly seen one
[f] from the beginning to the time point when the participant stated to have found all targets
