# Supplementary figures and images for: Exergames Encouraging Exploration of Hemineglected Space in Stroke Patients With Visuospatial Neglect: A Feasibility Study
Source: JMIR Serious Games. 2017 Aug 25;5(3):e17. doi: 10.2196/games.7923 (PMC5591404; doi:10.2196/games.7923)

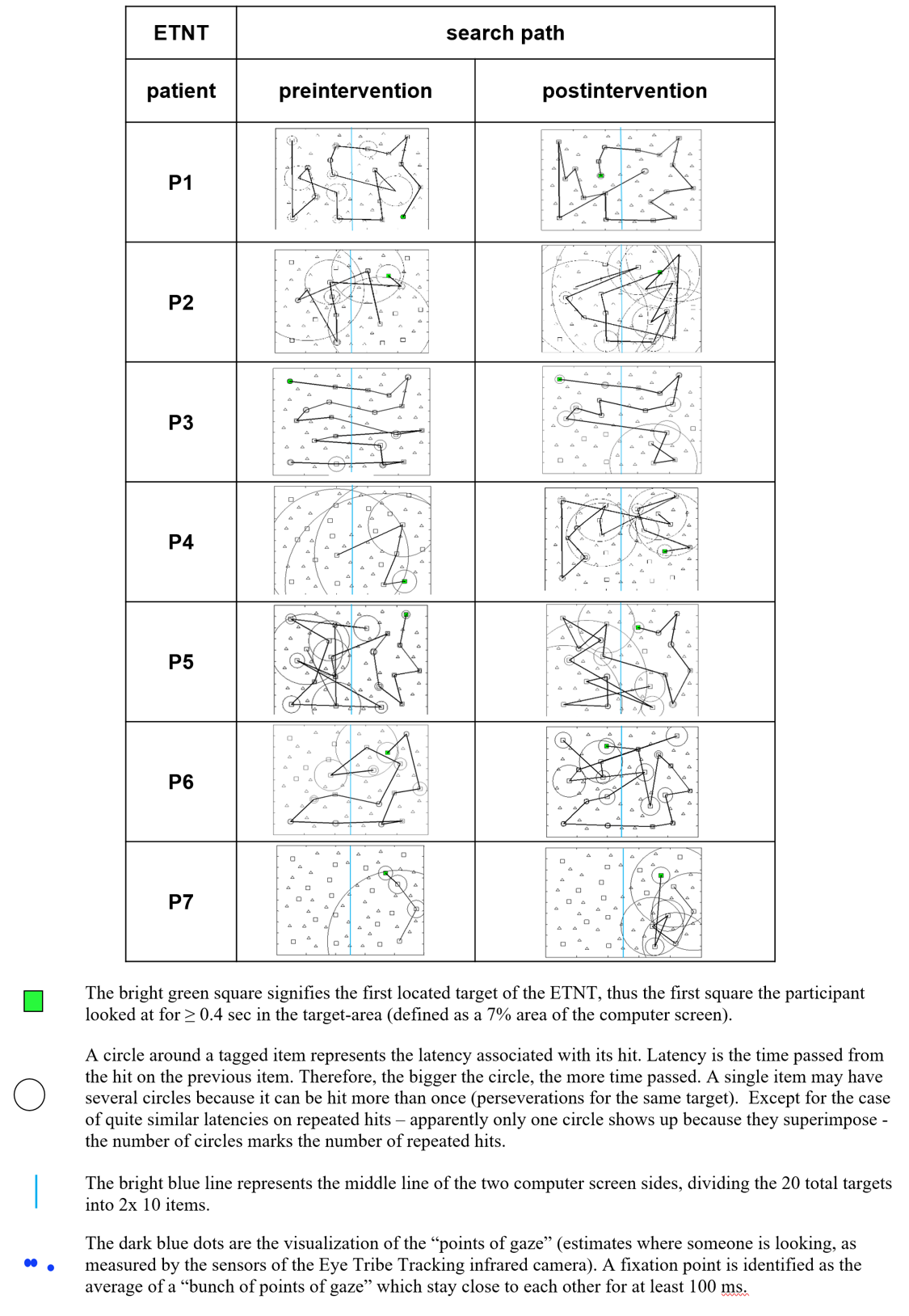

Supplement: Multimedia Appendix 3 [file games_v5i3e17_app3.png]

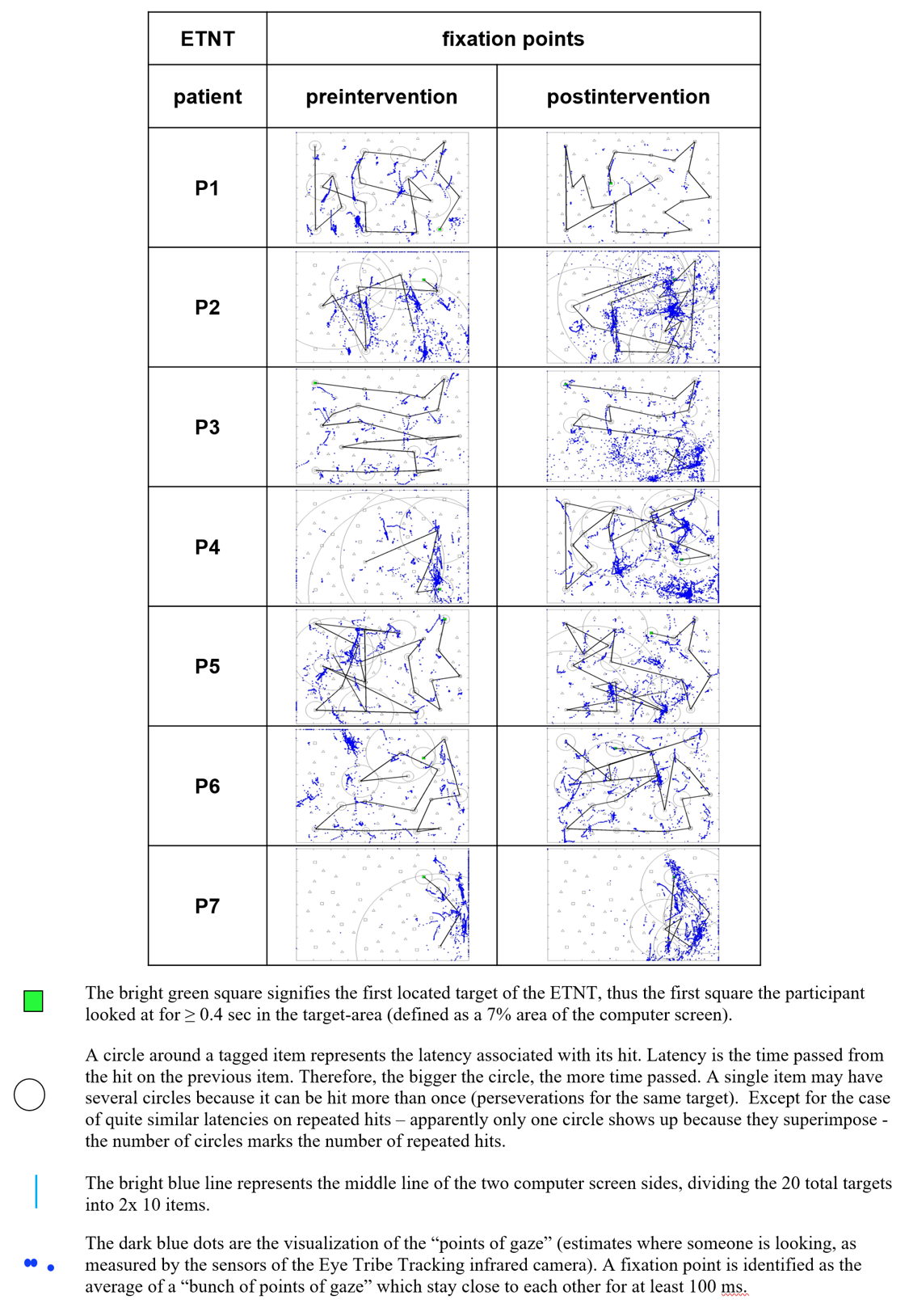

Supplement: Multimedia Appendix 4 [file games_v5i3e17_app4.png]

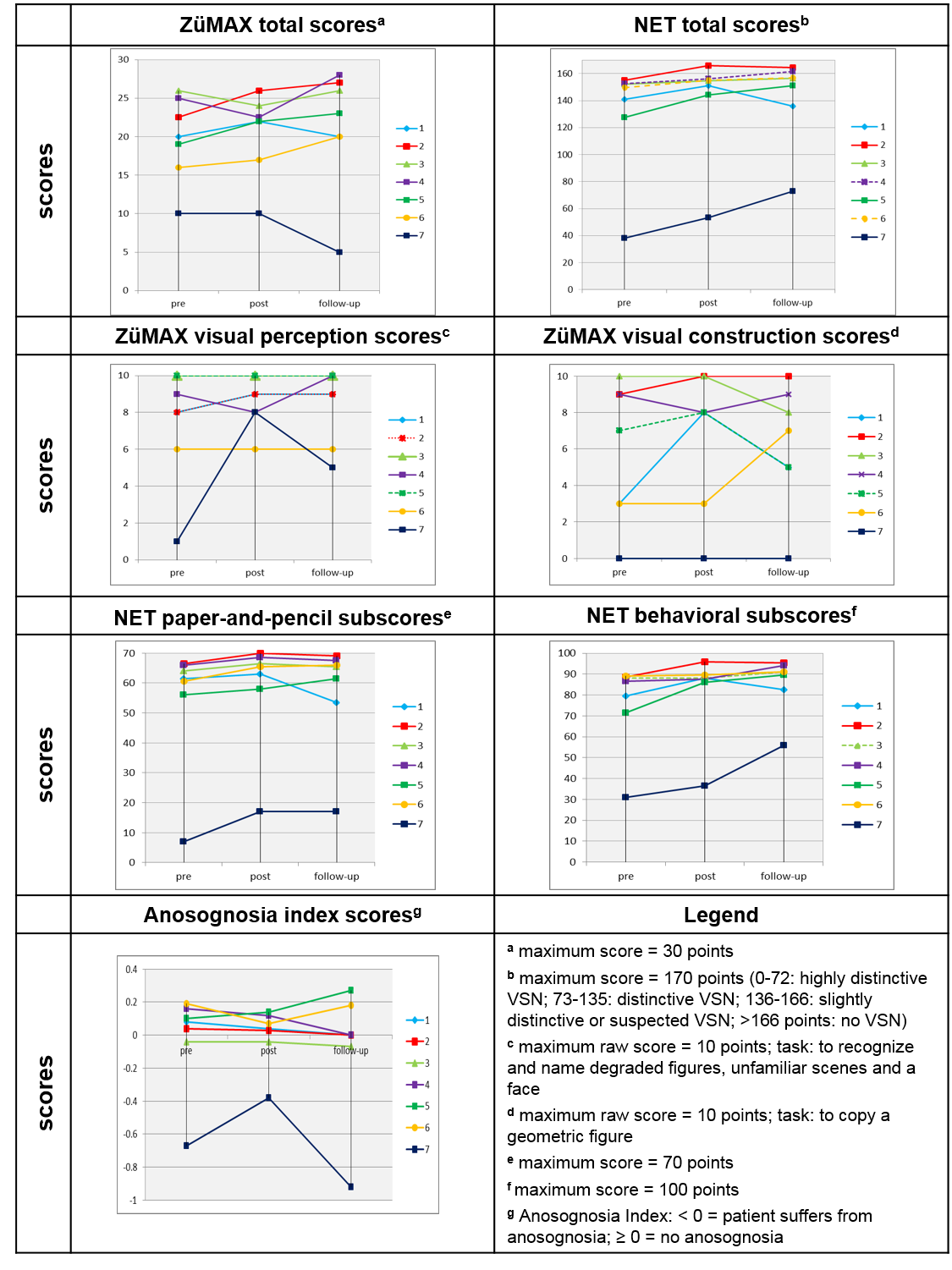

Supplement: Multimedia Appendix 6 [file games_v5i3e17_app6.png]
