# Supplementary material for: Exergames Encouraging Exploration of Hemineglected Space in Stroke Patients With Visuospatial Neglect: A Feasibility Study
Source: JMIR Serious Games. 2017 Aug 25;5(3):e17. doi: 10.2196/games.7923 (PMC5591404; doi:10.2196/games.7923)
Supplement: Multimedia Appendix 5 [file games_v5i3e17_app5.pdf]

mentassess-	intervention	IQR (25/50/75)			WSR							
		pre	post	FU	pre-post-FU		pre-post			post-FU		
					P	X^2	*P*	Z	r	*P*	Z	r
ZüMAX	total scores[a]	16<br>20<br>25	17<br>22<br>24	20<br>23<br>27	.29	2.48	.40	-0.84	-.32	.45	-0.76	-.29
	visual perception subtest[b]	6<br>8<br>10	8<br>9<br>10	6<br>9<br>10	.14	4.00	.26	-1.13	-.43	.66	-0.45	-.17
	visual construction subtest[b]	3<br>7<br>9	3<br>8<br>10	5<br>7<br>9	.64	0.90	.26	-1.13	-.43	.68	-0.41	-.16
NET	total scores[c]	127.5<br>149.5<br>152.5	144<br>154.5<br>156	136<br>156.5<br>161.5	.02*	8.00	.01*	-2.37	-.90[†]	.24	-1.19	-.45
	paper-and-pencil subtests[d]	56<br>61.5<br>66	58<br>65.5<br>68.5	53.5<br>65.5<br>67.5	.01*	8.67	.02*	-2.37	-.90[†]	.34	-0.95	-.36
	behavioral subtests[e]	71.5<br>86.5<br>88.5	86<br>88<br>89.5	82.5<br>91<br>94	.01*	10.30	.03*	-2.20	-.83[†]	.18	-1.35	-.51[†]
**Anosognosia Index[f]**		-0.04<br>0.08<br>0.16	-0.04<br>0.04<br>0.12	-0.07<br>0<br>0.18	.12	4.22	.75	-0.32	-.12	.50	-0.68	-.26

* = statistically significant; [†] large effect size (≥ 0.5)

AI = Anosognosia Index; FU = follow-up; IQR = Inter Quartile Range; NET = Neglect Test; *P* = level of significance ($P \leq 0.05$); r = effect size; VSN = visuo-spatial neglect; WSR = Wilcoxon signed rank test; Z = Z-score (approximation of the observed difference in terms of the standard normal distribution); ZüMAX = Zürich maxi mental status inventory

[a] maximum score = 30 points
[b] maximum raw score = 10 points
[c] maximum score = 170 points (0-72: highly distinctive VSN; 73-135: distinctive VSN; 136-166: slightly distinctive or suspected VSN; >166 points: no VSN)
[d] maximum score = 70
[e] maximum score = 100
[f] index < 0: patient suffers from anosognosia; index ≥ 0: no anosognosia
