# Supplementary material for: Exergames Encouraging Exploration of Hemineglected Space in Stroke Patients With Visuospatial Neglect: A Feasibility Study
Source: JMIR Serious Games. 2017 Aug 25;5(3):e17. doi: 10.2196/games.7923 (PMC5591404; doi:10.2196/games.7923)
Supplement: Multimedia Appendix 7 [file games_v5i3e17_app7.pdf]

test	ZüMAX								NET							
patient	pre-post-FU		pre-post			post-FU			pre-post-FU		pre-post			post-FU		
	P	X^2	*P*	Z	r	*P*	Z	r	*P*	X^2	*P*	Z	r	*P*	Z	r
**P1**	.61	1.00	.41	-0.82	-.31	.28	-1.10	-.42	.23	2.97	.06	-1.90	-.72†	.04*	-2.11	-.80†
**P2**	.10	4.67	.18	-1.34	-.51†	.32	-1.00	-.38	.07	5.33	.06	-1.86	-.70†	.41	-0.83	-.31
**P3**	.93	0.15	.66	-0.45	-.17	.41	-0.82	-.31	.54	1.23	.67	-0.42	.16	.92	-0.11	-.02
**P4**	.11	4.43	.36	-0.92	-.35	.11	-1.60	-.61†	.61	1.00	.45	-0.76	-.29	.29	-1.05	-.40
**P5**	.29	2.46	.28	-1.10	-.42	.79	-0.27	-.10	.01*	8.97	.14	-1.47	-.56†	.25	-1.16	-.44
**P6**	.10	4.67	.32	-1.00	-.38	.18	-1.34	-.51†	.24	2.88	.33	-0.99	-.37	.73	-0.34	-.13
**P7**	.06	5.69	.98	0	0	.06	-1.89	-.71†	.00*	14.11	.13	-1.52	-.58†	.11	-1.58	-.60†

* = statistically significant; † large effect size (≥ 0.5)

P = level of significance ($P \leq 0.05$); r = effect size; X^2 = Chi-Square; Z = Z-score (approximation of the observed difference in terms of the standard normal distribution)
